# Effects of Psychoeducational Interventions Using Mobile Apps and Mobile-Based Online Group Discussions on Anxiety and Self-Esteem in Women With Breast Cancer: Randomized Controlled Trial

**DOI:** 10.2196/19262

**Published:** 2021-05-18

**Authors:** Elaheh Ghanbari, Shahrzad Yektatalab, Manoosh Mehrabi

**Affiliations:** 1 Department of Mental Health and Psychiatric Nursing School of Nursing and Midwifery, Student Research Committee Shiraz University of Medical Sciences Shiraz Iran; 2 Department of Mental Health and Psychiatric Nursing School of Nursing and Midwifery, Community-Based Psychiatric Care Research Center Shiraz University of Medical Sciences Shiraz Iran; 3 Department of E-Learning Planning in Medical Sciences, Virtual School Shiraz University of Medical Sciences Shiraz Iran

**Keywords:** anxiety, breast cancer, education, mobile app, self-esteem

## Abstract

**Background:**

Psychoeducation has turned into an effective tool in taking care of cancer patients and improving their psychophysical symptoms and quality of life. Despite the growing use of mobile phone apps in medical settings for improving health, evidence supporting their effectiveness in the psychoeducation of patients with breast cancer is rarely available.

**Objective:**

This study was conducted to investigate the effect of psychoeducational interventions on anxiety and self-esteem in women with breast cancer using a mobile app and an online support group.

**Methods:**

An unblinded randomized controlled trial based on mobile phones was conducted in Shiraz, Iran. A research assistant recruited 82 women with nonmetastatic breast cancer aged 20 to 60 years were from clinics during a face-to-face visit at the point of care and randomly assigned to an intervention group (n=41) and a wait-list control group (n=41) through blocked randomization. The intervention group received psychoeducational interventions through a mobile phone app and participated in nurse-assisted online mobile support sessions for a total four weeks, whereas the control group was put on a waiting list. The State-Trait Anxiety Inventory (STAI) and the Rosenberg Self-Esteem Scale (RSES) were used to measure the levels of anxiety and self-esteem as the main outcomes at baseline and one week after the intervention.

**Results:**

A total of 82 patients with a mean age of 46.45 (SD 9.29) years recruited in Winter 2016 were randomly assigned to a wait-list control group (n=41) and intervention group (n=41). Five patients dropped out for different reasons. Comparing the postintervention mean scores of anxiety and its subscales using the independent t test showed statistically significant differences between the mobile psychoeducation group and controls (*P*<.001). The paired *t* test used to compare the postintervention mean scores of anxiety with its preintervention scores in the intervention group showed significant reductions in the scores of anxiety (95% CI –17.44 to –8.90, *P*<.001, *d*=1.02) and its two subscales (state anxiety: 95% CI –9.20 to –4.21, *P*<.001, *d*=0.88 and trait anxiety: 95% CI –8.50 to –4.12, *P*<.001, *d*=0.94). Comparing the postintervention mean scores of self-esteem showed statistically insignificant differences between the control and intervention groups (16.87 vs 17.97, *P*=.24). In contrast with the controls, using the paired *t* test showed that the increase in the postintervention mean scores of self-esteem were statistically significant in the intervention group compared with the preintervention scores (mean difference 2.05, 95% CI 1.28 to 2.82, *P*<.001).

**Conclusions:**

This study demonstrated the key role of mobile apps in decreasing anxiety and improving self-esteem in women with breast cancer through psychoeducational interventions. Similar studies with longer follow-ups are recommended that be conducted in this context.

**Trial Registration:**

Iranian Registry of Clinical Trials IRCT2015072123279N2; https://en.irct.ir/trial/19882

## Introduction

As the most prevalent cancer in women and the second most prevalent cancer, breast cancer accounts for 25.2% of cancers globally and 24.6% of cancers in Iran [1,2]. Advanced sciences, early diagnosis, and timely treatments have increased the survival rate of patients with breast cancer. The long-term survival of patients with breast cancer, however, predisposes them to many psychological disorders [3].

Psychological distress is highly prevalent among women with breast cancer, and they are at increased risks for developing anxiety. The prevalence of anxiety was found to be 24.1% in patients with breast cancer [4]. Research suggests higher levels of anxiety are associated with increased fear of recurrence, hopelessness, uncertainty, and loss of control and lower levels of satisfaction with life. Cancer-associated anxiety can intensify the disease symptoms in patients, prolong their recovery, cause undesirable outcomes, and degrade their and their caregivers’ quality of life [5,6]. Anxiety is the most common response to a mastectomy, potentially in association with body image, pain, and disfigurement, and is reported to be normally associated with a sense of disability that lowers self-esteem in women who have undergone a mastectomy [7,8].

Moreover, the changes caused by breast cancer and its treatment, mastectomy, changes in roles and functions, fear of losing femininity, sexual dysfunction, and impaired body image are considered major factors that lower self-esteem in patients [9]. Research has demonstrated that self-esteem was found to be adversely affected in women with breast cancer, which in turn can further impair their psychological well-being [10]. Moreover, low self-esteem was found to be associated with anxiety in this group of women [11].

High risks for developing anxiety and low self-esteem have been repeatedly reported in women with breast cancer. These patients therefore require special support in different stages, including diagnosis, treatment, rehabilitation, and follow-up [8,12].

Given the overwhelming effects of anxiety and low self-esteem on mental health and quality of life in women with breast cancer and their families and caregivers, effective psychological interventions (eg, psychoeducation and cognitive behavioral therapy) are recommended to be designed to improve quality of life and functions in these patients, shorten their recovery, and improve their prognosis [13,14]. Research suggests beneficial effects of psychoeducational interventions on patients with breast cancer [15,16].

Clinicians and patients have increasingly used mobile devices in the last decade. Given the growing use of mobile health apps and devices in medical sciences and the health care industry, the World Health Organization defined mobile health (mHealth) as using mobile and wireless technologies to help achieve health objectives [17]. mHealth apps include patient education, disease self-management, remote monitoring of patients, diagnostic treatment services, data collection, communication, and counseling services [18].

With a key role in the management and delivery of cancer care, mHealth can assist health care professionals and patients with the diagnosis of cancers and their associated psychological distress as well as follow-up, planning, providing cancer-related information, supporting medication adherence, and managing side effects [19].

Bender et al [20] reported hundreds of cancer-focused apps with the potential for conveniently providing real-time support interventions, monitoring a host of symptoms and physiological indicators of the disease, and promoting behavioral changes in a cost-effective manner; nevertheless, there is a lack of evidence for their safety and effectiveness, and little is known about interventions based on smartphones because many studies lacked a comparison or control group. Recently conducted studies support the effectiveness and efficacy of mobile and internet interventions [21,22]. Moreover, few standardized valid apps in the oncological field exist that can help with providing cancer care and supporting patients during their treatment and follow-up [23].

In addition to promoting health-related quality of life and decreasing stress, mHealth-based interventions were found to improve weight loss and physical activity in patients with breast cancer [24-26]; nevertheless, the evidence for the effects of mHealth apps and mental health interventions on psychological dimensions in patients with breast cancer is inconclusive and conflicting. According to Foley et al [27], acquiring information about breast cancer decreases anxiety levels before and after a breast cancer surgery; however, they reported higher anxiety levels after surgery in women who used mHealth apps compared with controls.

Although patients rated self-esteem as extremely high and very important in mHealth intervention, this dimension has not been included in previous psycho-oncological mHealth interventions [28].

Given that systematic reviews of mHealth apps in breast cancer are mainly limited to feasibility or pilot studies, the validity of their results is questionable [29]. To the best of our knowledge, randomized controlled trials (RCTs) have rarely addressed the effectiveness of mobile psychoeducational apps in self-esteem and anxiety in women with breast cancer given the large number of available mHealth apps. Although the efficacy of app-based interventions in reducing the symptoms of depression and anxiety has been demonstrated in RCTs, mHealth apps rarely take an expert approach, adhere to relevant medical evidence, or reflect patient needs. Research suggests using an app coupled with human support (eg, coaching and supervision through phone or text messages) can help increase engagement and use and promote the outcomes [30,31]; for instance, a mobile phone app supported with a behavioral health intervention based on social media was found to promote quality of life and psychosocial and physiological outcomes in breast cancer survivors within 10 weeks [32].

Given the positive effects of mHealth, this study developed an mHealth phone app known as the Breast Cancer Support zone (BCSzone) to help satisfy the needs of patients with breast cancer for psychoeducational interventions. This study aimed at exploring the effects of psychoeducational interventions on anxiety and self-esteem in women with breast cancer using the mobile app and mobile-based online group discussions. Our study hypothesis states that mobile-based psychoeducational interventions can improve self-esteem and reduce anxiety in patients with breast cancer.

## Methods

### Study Design

This RCT used a pretest and posttest design to investigate the impact of a mobile app-based psychoeducational intervention on anxiety and self-esteem in women with nonmetastatic breast cancer presenting to breast clinics in Shiraz, Iran. The patients were randomly assigned to the wait-list control group and intervention group receiving the psychoeducational interventions through the mobile phone app.

### Setting and Sample

In winter 2016, recruitment was performed in two breast clinics affiliated with Shiraz University of Medical Sciences, Shiraz, Iran, during clinic health appointments. Recruitment was achieved with posters and flyers about the study distributed in clinics, online announcements on the website of Shiraz University of Medical Sciences, and direct referrals by oncologists and health care providers at the study settings.

In the recruitment step, patients were briefed on the study objectives and procedure through face-to-face communication and provided with a brochure containing the aim and basic information about the study in the reception of the breast clinics and examination rooms. Clinicians at each clinic were furnished with information regarding the study, and the researchers also responded to the inquiries and concerns of the participants. After informed consent was obtained, participants completed all baseline questionnaires and received a 45-day package of free mobile data (50 GB) for their participation in the study.

The inclusion criteria comprised age range of 20 to 60 years, willingness to participate in the study, diagnosis of nonmetastatic breast cancer, literacy, access to smart mobile electronic devices connected to the internet and willingness to have the app installed on them, ability to work with the app and social networks, and moderate-to-severe anxiety (State-Trait Anxiety Inventory [STAI] score of >80 [33]) and low-to-moderate self-esteem (Rosenberg Self-Esteem Scale [RSES] score of <25 [34]). The exclusion criteria consisted of failing to regularly participate in the educational therapeutic program, serious disease other than breast cancer, history of chronic psychological disorders and taking psychiatric drugs, and participation in similar psychoeducational programs, which could have biased the results.

The minimum sample size was calculated as 25 per group in Number Cruncher Statistical System (NCSS) based on the data obtained by Aghabarari et al [35], mean anxiety difference score of 3 (SD 2.08), effect size of 0.79, significance level of .05, and test power of 80%. The ultimate sample size was calculated as 41 in each group considering the dropout effect.

**Figure 1:**
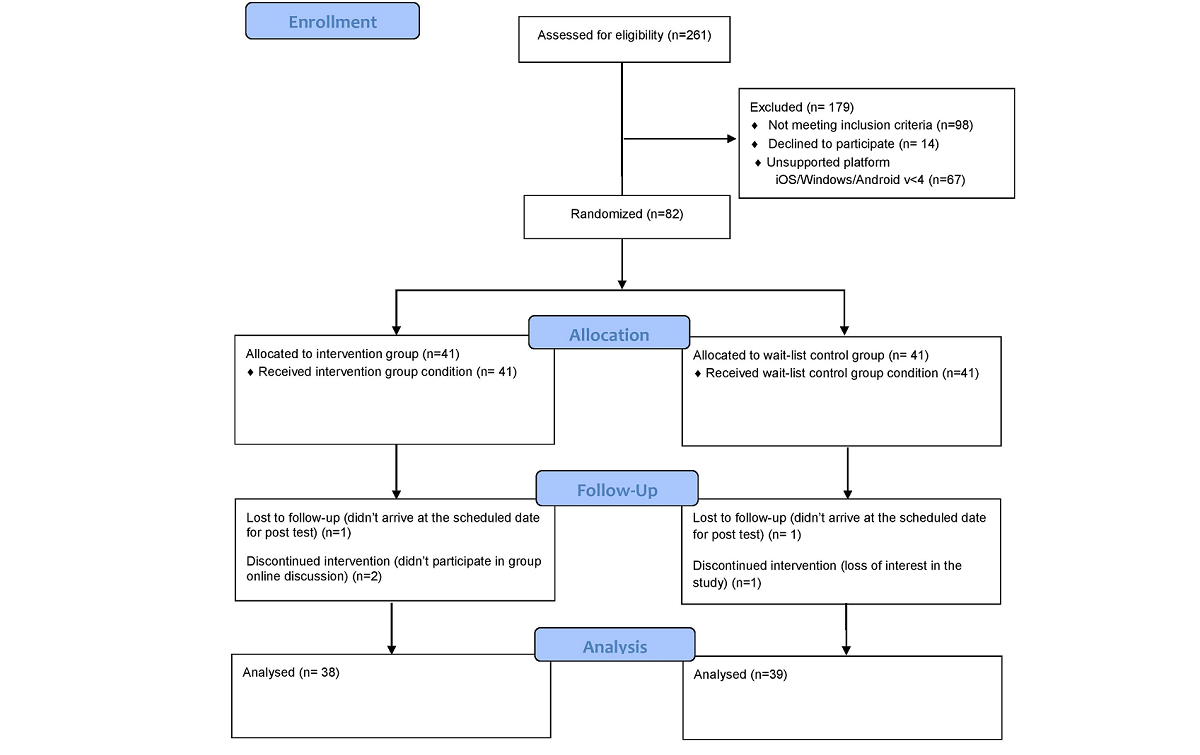
Consolidation Standards of Reporting Trials diagram.

In winter 2016, out of 261 women with breast cancer who volunteered to participate in the study, 82 were found to be eligible. Random Allocation Software (version 1.0.0, Saghaei M, Isfahan University of Medical Sciences, Iran) was used to generate a randomization schedule. After signing written informed consent forms, the women were randomly assigned to the intervention group (n=41) or the wait-list control (n=41); group randomization was performed by the researcher’s assistant. The study flow is illustrated in Figure 1.

As shown in Figure 1, randomization and attrition data were organized according to CONSORT (Consolidated Standards of Reporting Trials) guidelines, with 2 patients in the control group and 3 in the intervention group dropping out due to failure to complete the intervention (n=2), unwillingness to continue with the study (n=1), and lost to follow-up (n=2). The control group was put on a waiting list and only received routine health care, whereas the intervention group received psychoeducational interventions through a mobile app and participated in mobile-based online group discussions for 1 month. Pretest and posttest data were collected using paper-based instruments. The outcome assessors were different from the researchers who conducted and monitored the interventions.

### Ethical Considerations

This study was approved by the Ethics Committee of Shiraz University of Medical Sciences, Shiraz, Iran (IR.SUMS.REC.1395.20), and registered in the Iranian Registry of Clinical Trials [IRCT2015072123279N2] [36]. Written informed consent was obtained from all the study participants, and their voluntary participation in the entire process of the study and confidentiality of their information were ensured. All participants were allowed adequate time to carefully review the consent forms and ask relevant questions before signing them. At the end of the study, all patients in the wait-list control group also received the psychoeducational intervention.

### Outcome Measures

The data collection tools comprised the STAI, RSES, and demographic information questionnaires, including age, marital status, level of education, employment status, and history of mastectomy. The STAI and RSES were used to measure anxiety and self-esteem as the study variables and compare them in the 2 groups before and 1 week after the intervention. At the end of the treatment, the intervention group completed a satisfaction survey of the mobile-based intervention designed by the authors (Multimedia Appendix 1).

#### Anxiety

Anxiety was measured with the 40-item STAI as a self-report instrument that quantifies adult anxiety. The STAI comprises two 20-item subscales, state and trait anxiety. The state anxiety subscale evaluates the current anxiety status by determining how the respondent feels right now, whereas the trait anxiety subscale assesses how individuals generally and usually feel. Each item is scored on a 4-point Likert scale, resulting in a total subscale score of 20 to 80, with higher scores denoting higher anxiety levels. The total STAI score therefore equals 40 to 160 [37]. This valid and reliable questionnaire introduced by Spielberger et al [37] in 1970 has been translated to many different languages. An overall median Cronbach alpha of .86 to .92 has also confirmed the internal consistency of this instrument in normative samples [37-39]. A reliability coefficient of .89 to .90 was reported in the literature for STAI-T and .86 to .95 for STAI-S in diverse populations and cultures. A Cronbach alpha of .886 for trait anxiety and .846 for state anxiety also confirmed the internal consistency of the Persian version of the STAI [39,40].

#### Self-Esteem

The 10-item RSES was used to measure self-esteem on a 4-point Likert scale ranging from strongly agree=0 to strongly disagree=3. The total score is 0 to 30, with higher scores denoting higher self-esteem. Rosenberg reported a reliability coefficient of .77 and a validity coefficient of .82 for this scale. Administering the RSES in 53 nations, Schmitt et al [41] also confirmed the validity and reliability of this test. Furthermore, the RSES has been translated and adapted to different languages, including Persian. Validity and reliability of the Persian version of the RSES have been confirmed previously [42]. Alizade et al [43] reported a reliability coefficient of .73 and a validity coefficient of .74 in Iran. In another study, the Cronbach alpha coefficient of the Persian version of the scale was calculated as .84 [44]. In this study, Cronbach alpha was reported .70 for the baseline reliability coefficient of the scale.

#### Survey of satisfaction with the mobile-based intervention

This 16-item author-designed survey assessed the attitude and satisfaction of the participants with the designed mobile app. The item scores were based on respondent selections of very frequently, frequently, occasionally, rarely, and very rarely.

### Intervention

#### Instructional Design

In the first step, BCSzone was developed as an Android-based smartphone app that provided users with educational materials, including texts, images, animations, quizzes, audio files, and video clips, that demonstrated how to accurately perform the exercises. This app included educational topics and psychological and practical exercises and tests to be used offline by the users. Screenshots of the mobile app are available in Multimedia Appendix 2.

The instructional design of this mobile-based intervention was based on a model developed in a virtual school, the Comprehensive Center of Excellence for E-Learning in Medical Sciences, Shiraz University of Medical Sciences. This model was used to design multimedia-based education and comprised the following steps: planning and analysis, text authoring and design, production and preparation of every medium, developing scenarios, ultimately evaluating the educational material and metadata preparation and storage, and technical evaluation and presentation. All these steps focused on the stage evaluation to provide proper feedback for each stage [45,46]. The e-content was developed using Adobe Flash in predesigned formats and based on the standards of the virtual school. After evaluating, editing, and modifying the produced content in alpha and beta versions, the final version was approved and the content was then verified and published as an Android app.

BCSzone is a guided self-management psychoeducational app comprising 4 main chapters and 40 modules as follows:

- The Breast Cancer chapter includes topics such as introduction to breast cancer, diagnostic and treatment procedures, postmastectomy care, chemotherapy, radiation therapy, exercise, and proper diet.
- The Stress Management chapter addresses topics such as anxiety symptoms and stress complications, teaching the techniques of stress management and emotion management, thought stopping, diaphragmatic and conscious breathing, progressive muscle relaxation, and guided imagery.
- The Self-Esteem chapter addresses different methods of promoting self-esteem such as thoughts of self-confirmation, styles of establishing effective communication, and assertiveness.
- The Anger Management chapter addresses anger management techniques and problem solving.

The main page of the app includes keys to the image gallery, objectives, about us, and references, which could be selected by the user to switch between the pages and use the desired content of the app.

The objectives part of the app includes the topics and their duration in a way that users could get to know about the courses, their order, and the time required to learn the contents of each part. The keys “Start lesson” and “Exit” are included at the bottom of the main page of the app.

When the Start button is clicked, the lessons begin to be run in a multimedia format and a speaker’s voice is played to help users select the features of the app including stop, forward, backward, and the next or previous slide. Each topic contains pictures, texts, tables, audio files, and animations tailored to the content, and the users must correctly respond to a multiple-choice test at the end of each educational topic before being permitted to continue studying and observing future slides. Users are provided with feedback if they incorrectly answer the questions. Efforts were therefore made to turn the teaching/learning process into a dynamic and effective process.

#### Procedure

BCSzone was installed on the mobile phones of the participants in the intervention group, and they were individually taught to use the app. They were also provided with written instructions and compact discs containing the app. During the study, the intervention group was provided with a phone number to contact if they encountered any technical problems associated with BCSzone.

After the app was installed on participant smartphones, educational materials could be viewed in a multimedia format with a test at the end of each educational topic. Participants who could learn the contents and consequently pass the tests in each chapter were directed to a WhatsApp online chat group to interact with one another. As the most popular communication and social media platform in Iran, WhatsApp constitutes a smartphone-based instant messaging app that enables users to send voice, text, video, or location using group communication features. A psychiatric nurse followed up with the patients and provided them with support, advice, and guidance for about 60 minutes per weekend, if needed, through the same WhatsApp online group chat. As the only reminder, the patients were reminded by the psychiatric nurse to use the app during the weekly sessions.

As found in Paxling et al [47], the psychiatric nurse feedback was based on guidelines as follows: validating the work of the participants and reporting difficulties, problem solving and clarification on how to perform the treatment methods, promoting the tasks, reinforcing the progress, and encouraging work continuation. The intervention was used by the intervention group during 4 weeks. One week after the intervention, study participants were asked to recomplete the STAI and RSES. The control group on the waiting list received routine health care only and completed the questionnaires.

### Statistical Analyses

All completed questionnaires were coded, and the data collected were analyzed in SPSS (version 22, IBM Corp). The demographic variables were expressed using descriptive statistics, including mean, standard deviation, and frequency. Depending on the type of data, 1-way analysis of variance, Fisher exact test, independent *t* test, paired *t* test, or chi-square test were used to perform inferential analysis. Differences between the 2 groups were examined by analyzing the categorical data using the Pearson chi-square test or Fisher exact test, the continuous normally distributed data using the *t* test, and the nonnormally distributed data using the Wilcoxon Mann-Whitney test. The Kolmogorov-Smirnov test was also used to investigate the data normality. *P*<.05 was set as the level of statistical significance.

Given the normal distribution of the variables, the paired *t* test was used to compare changes in the mean scores of total anxiety, state anxiety, trait anxiety, and self-esteem in the women with breast cancer in each group between before and 1 week after the psychoeducational intervention. The 2 groups were also compared using the independent *t* test.

The effect size of the intervention was estimated at small for Cohen *d*=0.20, medium for Cohen *d*~0.50 and large for Cohen *d*≥0.80 [48]. In addition, no missing values were reported in the main outcomes.

## Results

### Recruitment and Follow-up

This trial was conducted between October 2016 and February 2017. Recruitment and baseline assessments were first performed for 3 months, the intervention for 4 weeks, and the postintervention assessments for 1 week after the intervention. In winter 2016, recruitment was performed face-to-face during the clinic’s health appointment in two breast clinics affiliated with Shiraz University of Medical Sciences, Shiraz, Iran. A total of 82 women with breast cancer were randomly assigned to the intervention or wait-list control groups of 41. The flowchart in Figure 1 shows the participants included in each group, the excluded ones, and those who dropped out and their reason of exclusion.

### Demographic Characteristics

The demographic characteristics for all study participants are displayed in Table 1. Results showed nonsignificant differences between the 2 groups in terms of level of education (Fisher exact test, *P*=.85), marital status (Fisher exact test, *P*=.69), history of mastectomy (chi-square test, *P*=.65), age (*P*=.66), and employment status (Fisher exact test, *P*=.82) before the intervention. The mean age of the participants was 46.45 (SD 9.29) years, that of the control group 46 (SD 8.80) years and that of the intervention group 46.9 (SD 9.83) years. The majority of the participants were married (66/82, 81%) and housewives (65/82, 79%).

**Table 1:** Frequency distribution of the demographic characteristics of the study groups.

Characteristics		Intervention group (n=41)	Control group (n=41)	Total (n=82)	*P* value
Age (years), mean (SD)		46.9 (9.83)	46 (8.80)	46.45 (9.29)	.66^a^
**Education level, n (%)**					.85^b^
	Middle school	19 (46)	22 (54)	41 (50)	
	Diploma	18 (44)	14 (34)	32 (39)	
	Associate degree	1 (2)	1 (2)	2 (2)	
	Bachelor’s degree	3 (7)	4 (10)	7 (9)	
**Marital status, n (%)**					.69^b^
	Single	2 (5)	3 (7)	5 (6)	
	Married	35 (85)	31 (76)	66 (81)	
	Widowed	3 (7)	5 (12)	8 (10)	
	Divorced	1 (2)	2 (5)	3 (4)	
**Job status, n (%)**					.82^b^
	Staff/worker	5 (12)	7 (17)	12 (15)	
	Retired	2 (5)	2 (5)	4 (5)	
	Student	0 (0)	1 (2)	1 (1)	
	Housewife	34 (83)	31 (76)	65 (79)	
**Mastectomy, n (%)**					.65^c^
	Yes	23 (56)	26 (63)	49 (60)	
	No	18 (44)	15 (37)	33 (40)	

^a^Independent sample *t* test.

^b^Fisher exact test.

^c^Chi-square test.

### Anxiety

The intervention and control groups were not statistically different at baseline in terms of the mean scores of total anxiety (*P*=.41), state anxiety (*P*=.35), and trait anxiety (*P*=.65). Statistically significant differences were, however, observed between the 2 groups 1 week after completing the intervention (*P*<.001). Comparing the postintervention mean score of anxiety with its preintervention mean score in the intervention group using the paired *t* test showed significant reductions in the total score of anxiety (95% CI –17.44 to –8.90, *P*<.001) and the scores of state anxiety (95% CI –9.20 to –4.21, *P*<.001) and trait anxiety (95% CI –8.50 to –4.12, *P*<.001), confirming the effectiveness of the psychoeducational intervention in alleviating anxiety in the intervention group (Table 2).

**Table 2:** Comparison of changes in the mean scores of anxiety and self-esteem in the women in the intervention and control groups before and after the intervention.

Variable			Pretest mean (SD)		Posttest mean (SD)	Mean differences (95% CI)	*P* value		*t* score		Effect size
**Anxiety (total)**											
	G1^a^	103.68 (14.73)		90.66 (13.84)		–13.02 (–17.44 to –8.90)	<.001	6.40		1.02	
	G2^b^	106.31 (16.36)		106.92 (15.94)		0.61 (–0.69 to 1.92)	.34	0.95		0.15	
**State anxiety**											
	G1	51.39 (10.16)		44.68 (8.26)		–6.71 (–9.20 to –4.21)	<.001	5.45		0.88	
	G2	53.54 (8.92)		53.62 (8.65)		0.07 (–1.00 to 1.15)	.88	0.14		0.02	
**Trait anxiety**											
	G1	52.29 (7.29)		45.97 (7.97)		–6.31 (–8.50 to –4.12)	<.001	5.83		0.94	
	G2	52.77 (9.23)		53.31 (8.89)		0.53 (–0.35 to 1.43)	.22	1.22		0.18	
**Self-esteem**											
	G1	15.92 (4.35)		17.97 (4.69)		2.05 (1.28 to 2.82)	<.001	5.39		0.87	
	G2	16.64 (3.68)		16.87 (3.48)		0.23 (–0.24 to 0.71)	.33	0.97		0.15	

^a^G1: intervention group.

^b^G2: control group.

### Self-Esteem

No statistically significant differences were observed in the baseline mean scores of self-esteem in the 2 groups (*P*=.82). Comparing the postintervention mean scores of self-esteem showed statistically nonsignificant differences between the control and intervention groups (16.87 vs 17.97, *P*=.24).

Table 2 shows significant differences in the mean score of self-esteem between the pretest and posttest in the intervention group (95% CI –2.82 to –1.28, *P*<.001) with a large effect size (*d*=0.87) compared with those in the control group (95% CI –0.71 to 0.28, *P*=.33, *d*=0.15). In other words, the increase observed in the self-esteem scores after the intervention was more significant in the intervention group (95% CI –2.82 to –1.28, *P*<.001), suggesting the effectiveness of the psychoeducational intervention in increasing self–esteem in the intervention group.

In the intervention group, the within-group effect size (*d*) of all the measures was large between the pretest and posttest.

### Satisfaction With the Mobile-Based Intervention

Table 3 presents the results of the survey conducted at the end of the study on the psychoeducational intervention performed in the intervention group using the mobile app, showing that 92% (35/38) of participants were very satisfied with the mobile educational app. They identified mobile app-based educational materials as cost-effective and had a great tendency to receive psychoeducational interventions through the mobile app rather than through in-person meetings. According to 92% (35/38) of participants, mobile educational apps could provide them with the required educational materials independently of time and place.

**Table 3:** Results of the survey of satisfaction with the mobile-based intervention.

Statement	Very frequently, n (%)	Frequently, n (%)	Occasionally, n (%)	Rarely, n (%)	Very rarely, n (%)
1. The mobile app meets my educational needs.	27 (70)	9 (24)	2 (5)	0 (0)	0 (0)
2. I am satisfied with the mobile app and how it was installed on my phone.	21 (55)	8 (21)	9 (24)	0 (0)	0 (0)
3. I tend to receive mobile-based psycho educational interventions rather than face-to-face meetings.	28 (74)	10 (26)	0 (0)	0 (0)	0 (0)
4. It’s easy for me to use educational mobile apps.	18 (47)	15 (40)	4 (11)	1 (3)	0 (0)
5. Multimedia contents used in the mobile app are appropriate.	33 (87)	4 (11)	1 (3)	0 (0)	0 (0)
6. I prefer to receive educational materials through mobile apps.	27 (71)	9 (24)	1 (3)	1 (3)	0 (0)
7. Psychoeducational intervention through mobile technologies is cost effective for me.	38 (100)	0 (0)	0 (0)	0 (0)	0 (0)
8. It’s easy for me to talk about the educational needs and personal issues that come with the disease through the online chat room.	19 (50)	8 (21)	4 (11)	5 (13)	2 (5)
9. Through the educational mobile app, I can access educational materials at anytime and anywhere.	35 (92)	3 (8)	0 (0)	0 (0)	0 (0)
10. I like to use mobile phones to share educational information about the disease and how to control it.	20 (53)	9 (24)	9 (24)	0 (0)	0 (0)
11. By using this mobile app, I feel I can contribute more in making decisions about self-care.	17 (45)	16 (42)	5 (13)	1 (3)	0 (0)
12. By using educational mobile health apps, I feel that I have more control over myself.	10 (26)	11 (29)	13 (34)	3 (8)	1 (3)
13. Getting educational materials through the mobile app about the disease and how to deal with complications is motivating me to take care of myself.	18 (47)	16 (42)	3 (8)	1 (3)	0 (0)
14. Getting information and tips about the disease and its complications through the mobile app helps save time.	37 (90)	4 (11)	0 (0)	0 (0)	0 (0)
15. I can get information and guidance on the illness and how to deal with it through the mobile app when health care and education services are not available.	36 (95)	2 (5)	0 (0)	0 (0)	0 (0)
16. I am satisfied with the educational mobile app in general.	35 (92)	3 (8)	0 (0)	0 (0)	0 (0)

## Discussion

### Principal Findings

To the best of our knowledge, this clinical trial pioneered the investigation of the effect of mobile-based psychoeducation on women with breast cancer. Our study hypothesis stated mobile-based psychoeducation can increase self-esteem and decrease anxiety in patients with breast cancer. The intervention group received the psychoeducational intervention using BCSzone and participated in nurse-assisted online mobile support sessions. The hypothesis suggesting decreases in anxiety scores was confirmed in the intervention group. After the first week of the intervention, the scores reported for state anxiety and trait anxiety in the intervention were statistically and significantly higher than in the control group.

These findings suggest the effectiveness of psychoeducational interventions through a mobile app (BCSzone) in reducing state anxiety and trait anxiety in women with breast cancer, which is consistent with literature on the effectiveness of psychoeducational interventions that focused on patients with breast cancer delivered via group, face-to-face, and/or video formats [14-16,49].

The positive results obtained cannot be explained by a single factor, as psychoeducational interventions encompass a wide range of trainings and mental behavioral skills and psychological techniques. The obtained results can be explained by the factors used in this study such as cognitive behavioral methods, including relaxation and adaptation skills, thought stopping, conscious breathing, guided imagery, and stress and emotional management, whose effectiveness in emotional disorders such as anxiety has been proven [50,51].

This study found a significant increase in self-esteem levels in the intervention group 1 week after the intervention compared with the baseline levels of self-esteem, although between-group analysis suggested nonsignificant increases in these levels in the control group. Research suggests patients with breast cancer tend to become socially isolated through limiting their own social relationships and losing their interest in group and social activities. These patients can degrade their self-esteem through social isolation, which manifests itself as failing to express their feelings, dissatisfaction with their appearance and changes in their performance, and a lack of self-confidence [3]. BCSzone addresses different self-esteem promotion methods and skills, including self-confirmation thoughts, effective communication, and assertiveness skills.

Research suggests promoting assertiveness skills and self-confirmation can assist the patients in improving their performance and social interactions [52]. Moreover, self-esteem can be enhanced by improving performance, interpersonal relationship skills, and self-satisfaction. It is also suggested that self-esteem and performance affect each other to some extent and self-esteem increases after achieving a goal. As embedded features of the mobile app, lessons on assertiveness, anger and stress management, and effective communication skills for women with breast cancer were also found to enhance their self-esteem [53].

Although between-group comparison suggested no statistically significant differences in self-esteem, the increase in self-esteem scores in the intervention group was not negligible after the intervention compared with baseline. As a complicated multidimensional disorder, low self-esteem can be treated using different interventions such as psychoeducation and cognitive behavioral therapy. Although the majority of these dimensions were discussed within the app, the limited duration of the intervention can explain the statistically nonsignificant difference between the groups.

In contrast to previous studies, this study used a smartphone app with multiple advantages to perform psychoeducational interventions. The effectiveness and efficacy of mobile and internet interventions have been recently supported in literature. These interventions were found more effective than conventional treatments and as effective as face-to-face therapies for a wide range of conditions such as depression and anxiety [21,22]. Nowadays, a significant amount of time is spent using smartphones daily, and mobile apps can be increasingly employed for improving health and managing diseases given their low cost, accessibility, and availability. All multimedia contents provided by smartphones such as video clips, text messages, and images can also assist users in improving their health [54]. Patients with cancer were found to be interested in using mobile technologies for managing their disease, and the potential of smartphones for improving patient awareness of emotional well-being and stress by delivering therapeutic interventions and reducing anxiety has been highlighted in literature [55]. Moreover, the advantages offered by online support groups compared with those offered by face-to-face groups include greater scheduling convenience and an increased access to care for those who were otherwise unable to participate in these groups due to their social anxiety, personal concerns, residence in remote areas, or general health status [56]. Recent reviews revealed that social networking apps can promote discussion, consultation, and collaboration among health care professionals and patients. This research states that online mobile-based systems enhance different psychosocial and quality of life outcomes and reduce anxiety levels in patients with breast cancer [32]. These findings are consistent with the literature, including an uncontrolled study by Attai et al [57] that reported a rise in perceived knowledge and reductions in anxiety levels in patients with breast cancer as a result of participating in a support group on Twitter.

Despite the large number of currently available telehealth and mHealth apps in oncological settings, to the best of our knowledge, the number of RCTs evaluating app effectiveness in women with breast cancer is limited. Evaluating the effect of an mobile app on women with breast cancer through an uncontrolled study, Kuijpers et al [58] reported significant improvements in the 3 dimensions of quality of life (role functioning, emotional-mental health, and physical functioning) over time. A simple app-based e-support program was also found to improve quality of life, self-efficacy, and symptom interference in women with breast cancer during their chemotherapy [59]. Moreover, a study by Zhu et al [60] showed the participants to perceive the program as effective in improving their knowledge, emotional well-being, and confidence.

Patients with breast cancer can remotely participate in interventions using platforms such as mobile devices. A pilot study by Lengacher et al [26] showed a 6-week mobile mindfulness‐based program including yoga, sitting and walking meditation, and body scan to alleviate the psychophysical symptoms of stress, state anxiety, and depression and enhance quality of life in breast cancer survivors. This program was therefore found to be acceptable and feasible given its clinical effects on the psychophysical symptoms.

Numerous studies have demonstrated the impact of mobile phones and apps on the provision of support for cancer survivors and the treatment, control, and prevention of diverse disorders and diseases [18,29,61]. In contrast, Zhu et al [59] reported the insignificant effects of mobile-based support programs on depression, anxiety, and social support in women with breast cancer undergoing chemotherapy. Similarly, Foley et al [27] found an mHealth app developed to provide ample information for patients with breast cancer to negatively affect them and exacerbate their anxiety after surgery, although they reported lower anxiety levels in members of the control group, who did not use the app. This reduction in anxiety was also found to be correlated with higher quality of life in the controls. The discrepancies in findings between this and previous research can be explained by differences in the study design, the mobile app employed, the method of teaching patients and their demographic details, and outcome measures.

Educational subjects and practical and psychological tests and exercises are integrated into BCSzone. Each module ends by providing users with feedback on their incorrect responses to the test questions, if any. MacDonald [62] found the possibility of interacting and receiving feedback from the therapist, different features of the apps and homework, and symptom distress tracking to be the factors that help enhance patient adherence to mHealth apps. The cyberspace relationship also helps patients with free expression of their personal problems and sharing of their experiences and feelings. Smartphone and mobile apps provide a great opportunity to discover new modalities of monitoring, treatment, and research into psychiatric and mental health conditions [63]. The promising platform provided by mobile apps helps women with breast cancer acquire knowledge and interact with peers and health care experts whenever and wherever they need to [20].

This research found the intervention based on BCSzone as a multicomponent psychoeducation mobile app and a nurse-administered online group to enhance self-esteem and decrease anxiety in patients with breast cancer.

These findings can be improved by employing diverse interventions, including psychiatric nurse feedback and participation in online group discussion. Research suggests advanced social media and communication technology can be used to increase the involvement of patients with cancer in diverse types of discussion and reflection on their own health [64]. In addition, mobile and web-based online support groups enable women with breast cancer to express psychophysical and sexual problems arising from their disease and easily share their feelings and experiences with therapists and other patients; otherwise, they would be hesitant to express their problems in face-to-face support groups [65]. In line with our research, a study by Winzelberg at al [56] found an internet support group to help reduce perceived stress, cancer-associated traumas, and depression in females with primary breast cancer.

Seeking to share information, feelings, and coping strategies was reported as the main inclination of patients with breast cancer. Online communities constitute reliable platforms for providing cancer survivors with opportunities to meet other survivors, exchange ideas, and receive support [66].

According to McGraw and Hall [67], serious security and privacy issues in mobile apps and telehealth systems serve as obstacles to the clinical and industrial applications of these systems; nevertheless, many populations, especially chronically ill patients, have reported that the beneficial effects of using telehealth systems and mHealth apps outweigh the risks [68]. This study included only the psychiatric nurse and study participants in a private WhatsApp group, and the messages were end-to-end encrypted to ensure privacy in sharing identifiable health data.

At the end of this study, the intervention group was asked to complete the author-designed survey assessing their opinions of and satisfaction with the mobile app. The results showed satisfaction with the mobile app in 92% of the cases. They all believed in the cost-effectiveness of receiving educational materials through mobile apps and were eager to electronically receive psychoeducational interventions through mobile phones rather than through personal meetings. They also identified the use of mobile educational apps as a new experience. Using BCSzone was therefore found to help with the stepwise care of patients with cancer.

In approximately half the cases, patients with cancer were recently found to be willing to send and receive data through an app supporting their oncological treatment and follow-ups [23]. In line with this research, a study on mobile-based education and interventions reported that 69.2% of the participants found mobile phones to be effective, simple, and immediate tools in their learning, 72.2% found mobile-aided learning to be a new experience, and 73.4% believed that the mobile-aided learning approach is learner-oriented and flexible in time, pace, and place. Educational outcomes can be improved through developing the essential functionality of certain teaching and learning techniques based on the unique features of mobile phones. Furthermore, mobile systems can be employed to raise patient levels of access to psychological support and remotely perform psychological interventions when patients are vulnerable [69,70].

### Practical Implications

These findings can be used in evidence-based practices to determine the efficacy and cost-effectiveness of interventions in improving patient outcomes. Nurses and other health professionals can employ mobile technology to provide a larger number of patients with care, counseling services, and trainings. Mobile educational apps are important for encouraging women with breast cancer to participate in self-care programs. Given the positive and useful results obtained from administering the psychoeducational intervention using mobile apps and mobile-based online group discussions, it is recommended that this intervention be used as a simple, cost-effective, and useful method in patients with breast cancer.

### Study Limitations

This study limitations included its short duration (4 weeks) given that psychoeducation should be regularly performed over a long period. Moreover, several health care workers and more follow-ups are required for evaluating the long-term effects of the mobile app. Further studies with longer follow-ups and larger samples are also recommended to be conducted in this context. Furthermore, this study failed to measure adherence to the mobile app and the length of use by the users.

### Conclusion

These findings are consistent with the results of previous studies on patient education and provision of counseling and support through mobile apps. BCSzone and the nurse-administered online group discussion were found to reduce anxiety levels and raise self-esteem scores in women with breast cancer. This research highlighted the key role of using mobile apps in psychoeducational interventions in decreasing anxiety and improving self-esteem in women with breast cancer. Further studies are recommended that be conducted in this context using longer follow-ups.

## Appendix

Multimedia Appendix 1 Survey of Satisfaction with the Mobile-Based Intervention.

Multimedia Appendix 2 Screenshots of the mobile app (BCSzone).

Multimedia Appendix 3 CONSORT-EHEALTH checklist V1.6.

## Abbreviations

BCSzone: Breast Cancer Support zone
CONSORT: Consolidated Standards of Reporting Trials
mHealth: mobile health
RCT: randomized controlled trial
RSES: Rosenberg Self-Esteem Scale
STAI: State-Trait Anxiety Inventory
