# Supplementary material for: Effects of Psychoeducational Interventions Using Mobile Apps and Mobile-Based Online Group Discussions on Anxiety and Self-Esteem in Women With Breast Cancer: Randomized Controlled Trial
Source: JMIR Mhealth Uhealth. 2021 May 18;9(5):e19262. doi: 10.2196/19262 (PMC8170553; doi:10.2196/19262)
Supplement: Multimedia Appendix 1 [file mhealth_v9i5e19262_app1.pdf]

Supplemental material

Survey of Satisfaction with the Mobile-Based Intervention

Please put a check mark in the appropriate column for each of the following statements.

Statement	Very frequently	Frequently	Occasionally	Rarely	Very rarely
1. The mobile app meets my educational needs.					
2. I am satisfied with the mobile app and how it was installed on my phone.					
3. I tend to receive mobile-based psycho educational interventions rather than face-to-face meetings.					
4. It’s easy for me to use educational mobile apps.					
5. Multimedia contents used in the mobile app are appropriate.					
6. I prefer to receive educational materials through mobile apps.					
7. Psycho-educational intervention through mobile technologies is cost effective for me.					
8. It’s easy for me to talk about the educational needs and personal issues that come with the disease through the online chat room.					
9. Through the educational mobile app, I can access educational materials at anytime and anywhere.					
10. I like to use mobile phones to share educational information about the disease and how to control it.					
11. By using this mobile app, I feel I can contribute more in making decisions about self-care.					
12. By using educational mobile health apps, I feel that I have more control over myself.					
13. Getting educational materials through the mobile app about the disease and how to deal with complications is motivating me to take care of myself.					
14. Getting information and tips about the disease and its complications through the mobile app helps save time.					
15. I can get information and guidance on the illness and how to deal with it through the mobile app when health care and education services are not available.					
16. I am satisfied with the educational mobile app in general.					
