# Supplementary figures and images for: Effects of Psychoeducational Interventions Using Mobile Apps and Mobile-Based Online Group Discussions on Anxiety and Self-Esteem in Women With Breast Cancer: Randomized Controlled Trial
Source: JMIR Mhealth Uhealth. 2021 May 18;9(5):e19262. doi: 10.2196/19262 (PMC8170553; doi:10.2196/19262)

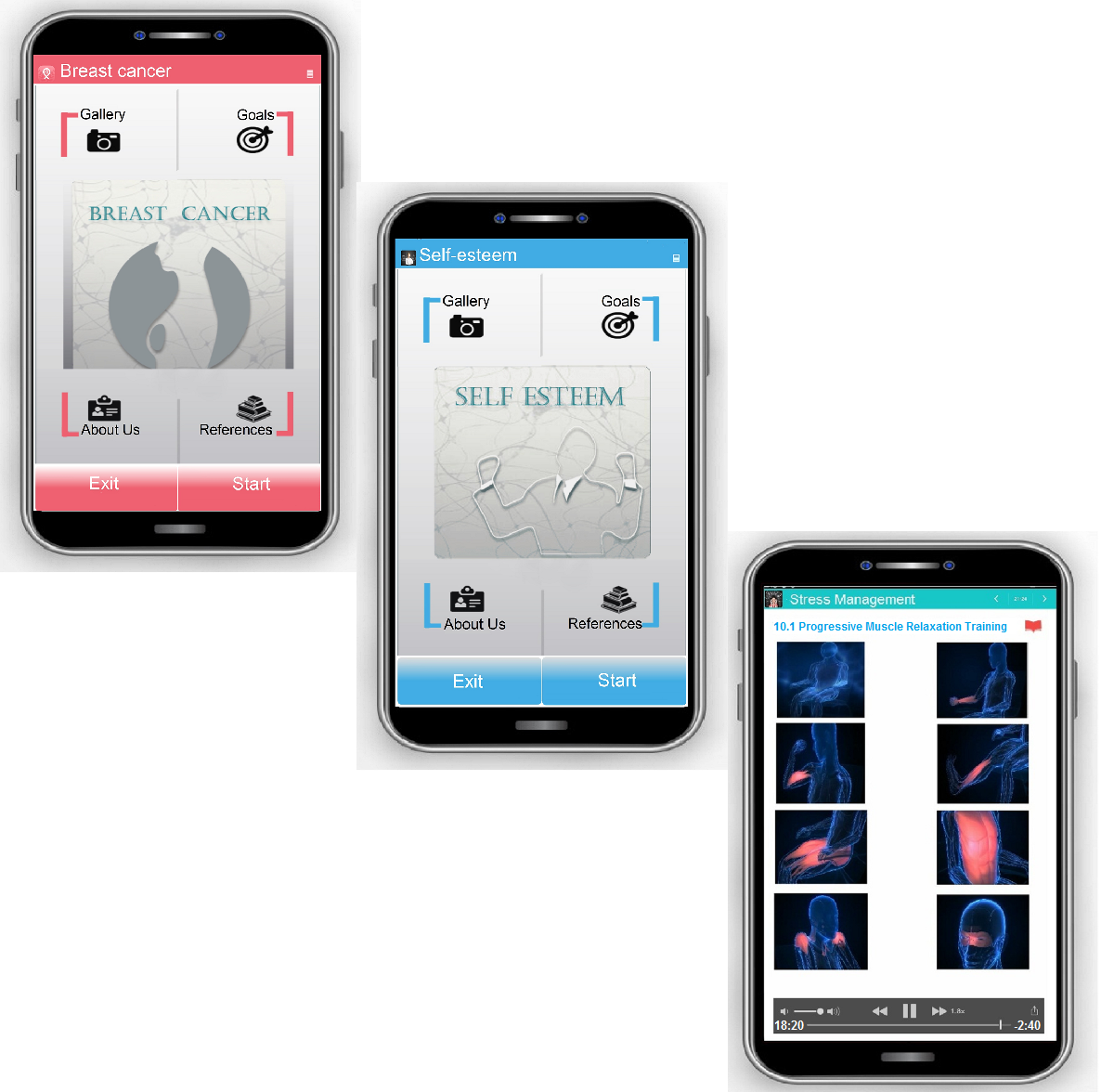

Supplement: Multimedia Appendix 2 [file mhealth_v9i5e19262_app2.png]
